# Effect of ventilation rate on recovery after cardiac arrest in a pediatric animal model

**DOI:** 10.1371/journal.pone.0237736

**Published:** 2020-08-20

**Authors:** Jorge López, Patricia Arias, Beatriz Domenech, Daniel Horcajo, Juan Pablo Nocete, Laura Zamora, Sarah Nicole Fernández, Jesús López-Herce

**Affiliations:** 1 Pediatric Intensive Care Department, Gregorio Marañón General University Hospital, Madrid, Spain; 2 Gregorio Marañón Health Research Institute, Madrid, Spain; 3 Mother-Child Health and Development Network (RedSAMID) of Carlos III Health Institute, Madrid, Spain; 4 School of Medicine, Complutense University of Madrid, Madrid, Spain; University of Alberta, CANADA

## Abstract

**Aims:**

To assess the impact of two different respiratory rates in hemodynamic, perfusion and ventilation parameters in a pediatric animal model of cardiac arrest (CA).

**Methods:**

An experimental randomized controlled trial was carried out in 50 piglets under asphyxial CA. After ROSC, they were randomized into two groups: 20 and 30 respirations per minute (rpm). Hemodynamic, perfusion and ventilation parameters were measured 10 minutes after asphyxia, just before ROSC and at 5, 15, 30 and 60 minutes after ROSC. Independent medians test, Kruskal-Wallis test and χ2 test, were used to compare continuous and categorical variables, respectively. Spearman’s Rho was used to assess correlation between continuous variables. A p-value <0.05 was considered significant.

**Results:**

Arterial partial pressure of carbon dioxide (PaCO2) was significantly lower in the 30 rpm group after 15 minutes (41 vs. 54.5 mmHg, p <0.01), 30 minutes (39.5 vs. 51 mmHg, p < 0.01) and 60 minutes (36.5 vs. 48 mmHg, p = 0.02) of ROSC. The percentage of normoventilated subjects (PaCO2 30–50 mmHg) was significantly higher in the 30 rpm group throughout the experiment. pH normalization occurred faster in the 30 rpm group with significant differences at 60 minutes (7.40 vs. 7.34, p = 0.02). Lactic acid levels were high immediately after ROSC in both groups, but were significantly lower in the 20 rpm group at 30 (3.7 vs. 4.7 p = 0.04) and 60 minutes (2.6 vs. 3.6 p = 0.03).

**Conclusions:**

This animal model of asphyxial CA shows that a respiratory rate of 30 rpm is more effective to reach normoventilation than 20 rpm in piglets after ROSC. This ventilation strategy seems to be safe, as it does not cause hyperventilation and does not affect hemodynamics or cerebral tissue perfusion.

## Introduction

Pediatric cardiac arrest (CA) is different from adult CA [1–3]. While adult CA is usually of cardiac origin, in children it is the result of asphyxia in the majority of cases [4–8]. Therefore, ventilation during cardiopulmonary resuscitation (CPR) is more important in children than in adults [9–11].

International CPR guidelines recommend using low respiratory rates (RR) during CPR in children [1,2]. However, the results of some experimental studies [12] and those of a recent clinical study [13] suggest that higher RR achieve better results in terms of survival.

There are only a few studies concerning ventilation strategies after the return of spontaneous circulation (ROSC) in adult and pediatric patients, and results are discordant. [1,2,9,14].

Both hypercapnia (arterial partial pressure of carbon dioxide -PaCO2-> 50 mmHg) and hypocapnia (PaCO2 <30 mmHg) 60 minutes after ROSC are associated with poor neurological outcomes and increased mortality in children [4,9,15]. However, achieving normoventilation immediately after ROSC is easier said than done in the clinical setting [9,16].

The objective of this study was to compare the effects of two different RR (20 and 30 respirations per minute -rpm-) after ROSC on ventilation, hemodynamics, and cerebral and tissue perfusion in a pediatric animal model of asphyctic CA.

## Materials and methods

An experimental, prospective, randomized controlled trial was conducted with 50 Maryland healthy and genetically identical piglets between 1 and 2 months of age, weighing between 7.5 and 15 kg. This animal model has been widely used for CA studies [12,17]. The study was conducted by accredited staff and international recommendations about animal care and animal experimentation and ARRIVE guidelines were followed. The experimental protocol was approved by the Gregorio Marañon University Hospital Ethics Committee for Animal Research. Investigators were not blind to the RR group.

### Experimental protocol

Animals were housed for at least 24 hours before the study. They were fasted overnight allowing free access to water. Piglets were pre-medicated with intramuscular ketamine (15 mg/kg) and atropine (0.02 mg/kg). After canalization of a peripheral vein in the ear, animals were anesthetized with boluses of propofol (5 mg/kg), fentanyl (5 μg/kg) and atracurium (0.5 mg/kg) for oral endotracheal intubation. Sedation and muscle relaxation (propofol 10 mg kg^-1^ h^-1^, fentanyl 10 mcg kg^-1^ h^-1^, and atracurium 2 mg kg^-1^ h^-1^) were maintained throughout the procedure to ensure comfort and avoid suffering.

Animals were mechanically ventilated with a Servo 900C^®^ respirator (Siemens-Elema, Solna, Sweden) with the following settings: 20 rpm, tidal volume (tV) of 10 ml/kg and an inspired oxygen fraction (FiO2) of 45%. Ultimate adjustments were made to achieve a PaCO2 between 35–45 mmHg.

An S5^®^ monitor (DatexOhmeda, Madison, Wisconsin, USA) was used for cardiorespiratory monitoring, including electrocardiogram, circuit pressures and volumes, as well as end-tidal CO2. A three-lumen 5F catheter was used for continuous central venous pressure (CVP) monitoring, blood sample extraction and drug infusion. Peripheral oxygen saturation (SatO2) and cerebral and splanchnic regional oxygen saturation (rSO2) were also monitored using HeartStart XL+^®^ monitor (Philips Medical Systems, Andover, Massachusetts, USA) and near-infrared spectroscopy (INVOS^®^ Cerebral Oxymeter Monitor, Somanetics, Troy, Minnesota, USA), respectively. Cerebral blood flow was measured by means of a flow sensor on the carotid artery (HDQ1.5FSB, Transonic Systems Inc., Ithaca, New York, USA). A tissue blood flow laser doppler sensor was placed on the abdomen to measure tissue perfusion (BLF21A Laser Doppler 121 Perfusion Monitor^®^, Transonic Systems Inc., Ithaca, New York, USA). Blood gases were analyzed using the GEM Premier 3000^®^ device (Instrumentation Laboratory, Lexington, USA).

After a 30-minute stabilization period, asphyxial CA was induced, as described in previous studies from our research work [12,17]. After confirming CA (defined as the presence of a mean arterial blood pressure (MBP) <25 mmHg), advanced CPR was delivered according to the international guidelines [1,2]. CPR was discontinued once ROSC was achieved or after 24 minutes of advanced CPR. After ROSC, piglets were randomly allocated into 2 ventilation groups: 20 or 30 rpm. The remaining respiratory settings were fixed at a FiO2 of 50% and a tV of 10 ml/kg. Animals were monitored for one hour after the ROSC, and were subsequently euthanized by administering supra-anesthetic doses of fentanyl and propofol immediately followed by a rapid intravenous infusion of potassium chloride (4 mEq/kg). Animals were allocated into the study groups by a computer generated sequence of random numbers (http://www.randomization.com). Random block sizes were used (10 subjects randomized into 5 blocks) to prevent disparity between groups in the case of premature study discontinuation.

The following variables were registered: heart rate -HR-, systolic blood pressure -SBP-, diastolic blood pressure -DBP-, MBP, CVP, arterial pH -pHa-, PaCO2, arterial oxygen partial pressure -PaO2-, bicarbonate -HCO3- and perfusion parameters such as cerebral and splanchnic rSO2, tissue and cerebral blood flow and arterial lactate. These variables were collected at baseline, 10 minutes after asphyxia, just before ROSC and 5, 15, 30 and 60 minutes after ROSC.

### Statistics

A sample size and power calculator was used to calculate sample size (GRANMO version 7.12 (Instituto Municipal de Investigación Médica, Barcelona, Spain). An arcsin approximation was applied to the two-sided test, accepting an alpha risk of 0.05 and a beta risk of 0.2. An estimated sample size of 25 subjects per group would allow statistical tests to detect statistically significant proportion differences of 40% in ventilation parameters (drop-out rate of 5%). Under these conditions, the calculated statistical power was 83%.

IBM SPSS Statistics 21.0 package was used for data analysis (IBM SPSS Statistics, Chicago, Michigan, USA). The Kolmogorov-Smirnov test revealed that variables did not follow a normal distribution. Continuous variables were expressed as medians (interquartile range -IQR-) and categorical variables with absolute numbers (percentages). The Kruskal-Wallis test (or the χ2 or Fisher test, if sample size was less than 20, or any value less than 5) were used to compare continuous and categorical values. Spearman’s Rho test was used to assess correlation between continuous variables. A p value <0.05 was considered significant.

## Results

Fifty piglets were included in the study. Median weight was 11 kg (9.4–12 kg) and median height was 72 cm (68–76 cm). Median time to CA was 8.1 minutes (7.1–8.5 minutes). Median time from the beginning of CPR until ROSC was 3 minutes (3–3.5 minutes).

No differences were found between groups at baseline or before ROSC except in cerebral rSO2 10 minutes after asphyxia 26% (15–37%) in the 20 rpm group vs 15% (15–17,5%) in the 30 rpm group; p = 0,03). (Table 1).

**Table 1:** Hemodynamic, perfusion and ventilation parameters immediately before ROSC. Comparison between groups.

Variables	20 rpm	30 rpm	p-value
SBP (mmHg)	92 (52.7–114.7)	93 (63–164)	0.85
DBP (mmHg)	31.5 (17.2–42)	32 (16.5–92)	0.74
MBP (mmHg)	41 (23.5–70)	36 (26–128)	1
CVP (mmHg)	12 (8.5–14.7)	12 (8.75–17.5)	0.70
Brain rSO2 (%)	41 (16.5–52.7)	44 (19–61)	0.68
Splanchnic rSO2 (%)	35 (28–44.2)	40 (27–42)	0.27
Carotid blood flow (L/min)	26 (7–74)	56 (8.5–88.5)	0.38
Tissue blood flow (L/min)	25.3 (20.3–33.5)	25.2 (17.5–37.4)	0.85
Temperature (°C)	37.2 (36.6–38.1)	37 (36.7–37.4)	0.17
Arterial lactate (mmol/L)	6.4 (6.2–7.0)	6.9 (6.2–7.4)	0.30
pHa	7.12 (7.11–7.2)	7.18 (7.1–7.27)	0.48
PaCO2 (mmHg)	70 (62–80.5)	62.5 (49–74.5)	0.40
PaO2 (mmHg)	68 (53–90.5)	60 (44–105.2)	0.87
HCO3 (mmol/L)	20 (18.6–21.3)	20.6 (17.9–21.4)	0.92

Data are expressed as medians (IQR)

ROSC: return of spontaneous circulation; rpm: respirations per minute; SBP: systolic blood pressure; DBP: diastolic blood pressure; MBP: mean blood pressure; CVP: central venous pressure; rSO2: regional oxygen saturation; pHa: arterial pH; PaCO2: CO2 arterial pressure; PaO2: O2 arterial pressure; HCO3: bicarbonate; IQR: interquartile range.

**Fig 1:**
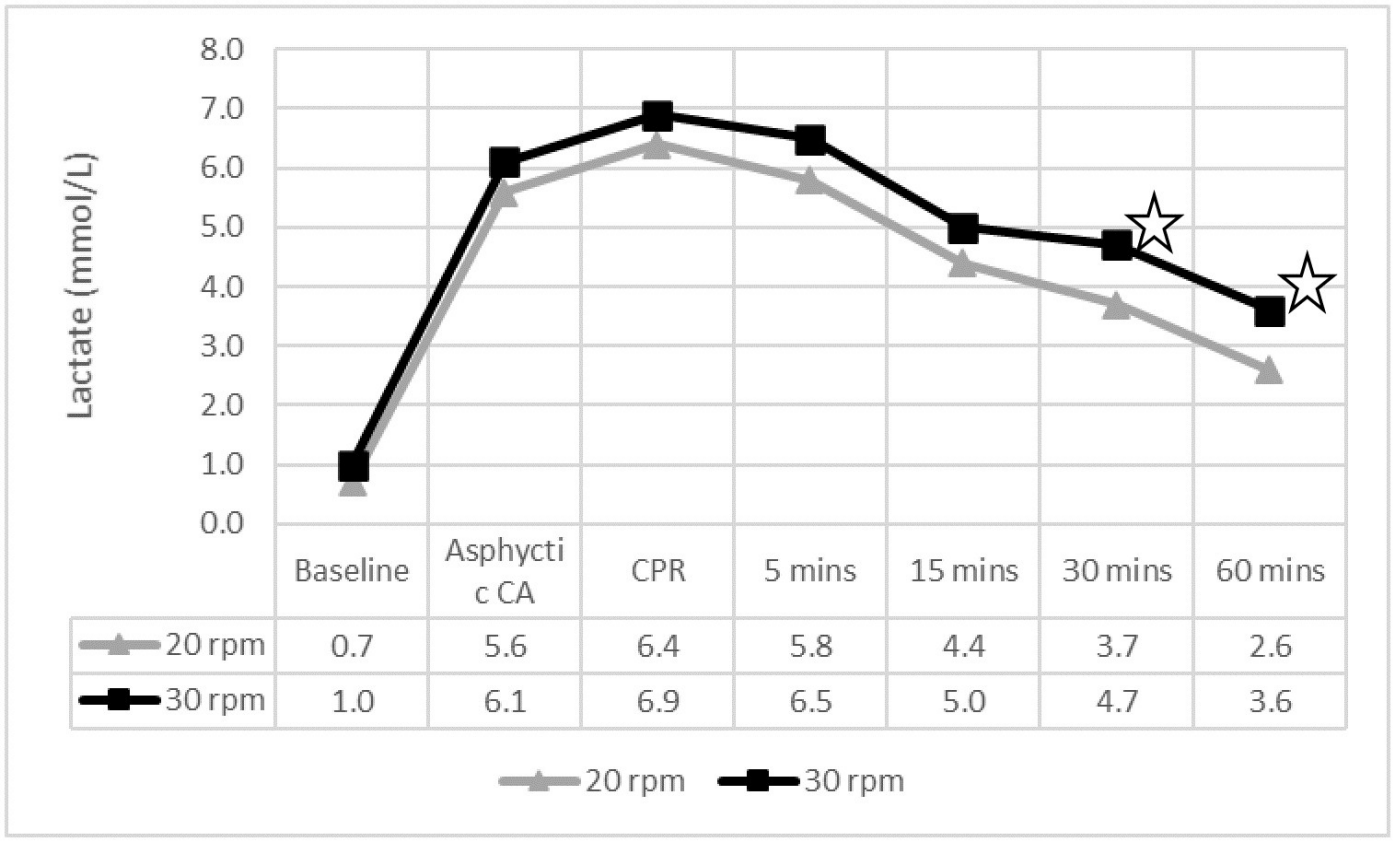
Lactate levels throughout the experiment. ✩ p < 0.05. CA: cardiac arrest; CPR: cardiopulmonary resuscitation; mins: minutes. 5 mins to 60 mins: time after ROSC.

After ROSC, lactic acid levels (at 30 and 60 minutes) and cerebral rSO2 (at 5 minutes) were significantly lower, and tissue blood flow (at 60 minutes) was significantly higher in the 20 rpm group, as shown in Fig 1, Tables 2 and 3.

**Table 2:** Hemodynamic parameters after ROSC.

Variables	Time	20 rpm	30 rpm	p-value
HR (bpm)	5 min	204 (199–212)	203 (192–210)	0.78
	15 min	183 (167–192.5)	177 (154–192)	0.57
	30 min	162 (142.5–176)	165 (139.5–183)	1
	60 min	174 (148.5–190.5)	156 (130–179.7)	0.47
SBP (mmHg)	5 min	173 (144.5–184)	167 (142.5–184.5)	0.40
	15 min	128 (100.5–161)	122 (82–139)	1
	30 min	101 (80.5–128)	103 (84.5–119.5)	1
	60 min	114 (93.5–137.5)	114 (85.5–132.2)	0.89
DBP (mmHg)	5 min	105 (76.5–116)	96 (74–121)	0.57
	15 min	71 (53–94)	64 (30–77.5)	1
	30 min	47 (38.5–67)	53 (33–64.5)	0.57
	60 min	64 (45.5–89)	60 (49–76.7)	0.88
MBP (mmHg)	5 min	130 (100.5–142.5)	116 (97–144)	0.57
	15 min	91 (71–117.5)	82 (44–98.5)	0.57
	30 min	66 (57–89.5)	73 (47.5–83.5)	1
	60 min	83 (68–99)	81 (61.2–91.2)	0.87
CVP (mmHg)	5 min	6.5 (4.2–9)	7 (1.5–10)	0.90
	15 min	5 (3–9)	3 (1–8.5)	1
	30 min	4.5 (3–7.5)	3 (2–8)	0.89
	60 min	4.5 (4–7)	4.5 (2–8.7)	0.77

Data are expressed as medians (IQR)

ROSC: return of spontaneous circulation; rpm: respirations per minute; HR: heart rate; BPM: beats per minute; SBP: systolic blood pressure; DBP: diastolic blood pressure; MBP: mean blood pressure; CVP: central venous pressure; IQR: interquartile range.

**Table 3:** Perfusion parameters after ROSC.

Variables	Time	20 rpm	30 rpm	p-value
Brain rSO2 (%)	5 min	62 (53–75.5)	69 (60–81)	**0.03**
	15 min	61 (51–70.5)	71 (59–79)	0.23
	30 min	61 (51–68.5)	64 (56.75–77.5)	0.87
	60 min	54.5 (49.7–66.7)	56.5(44.5–72.5)	0.75
Splanchnic rSO2 (%)	5 min	57 (49–66)	54 (47–66)	0.38
	15 min	63 (57–68)	59.5 (50.7–69.2)	0.88
	30 min	64 (59–69)	61 (53–65)	0.38
	60 min	62.5 (52–65.2)	57.5 (49.5–62.5)	0.37
Carotid blood flow (L/min)	5 min	130 (107–143)	133 (92–153)	0.65
	15 min	98 (60–113)	86 (53–102)	0.55
	30 min	62.5 (58.7–81)	63 (50–84)	0.89
	60 min	59.5 (48.7–71.5)	54.5 (34.5–63.2)	0.76
Tissue blood flow (L/min)	5 min	17 (9.3–22.8)	13.5 (9.2–27.3)	0.67
	15 min	12.9 (9.3–17.6)	10.2 (6–20.9)	0.77
	30 min	9.6 (8.4–14.7)	8.5 (5.7–13.2)	0.77
	60 min	10.6 (7.6–13.9)	7 (5.4–9.7)	**0.03**
Temperature (°C)	5 min	36.8 (36.2–37.4)	36.6 (36.1–37.1)	1
	15 min	36.8 (36.1–37.6)	36.6 (36.1–37.05)	0.40
	30 min	36.8 (36.2–37.6)	36.6 (36.1–37.0)	0.26
	60 min	37.1 (36.2–38.2)	36.8 (36.1–37.4)	0.47

Data are expressed as medians (IQR). Statistically significant values (p <0.05) appear in bold type.

ROSC: return of spontaneous circulation; rpm: respirations per minute; rSO2: regional oxygen saturation; IQR: interquartile range.

**Fig 2:**
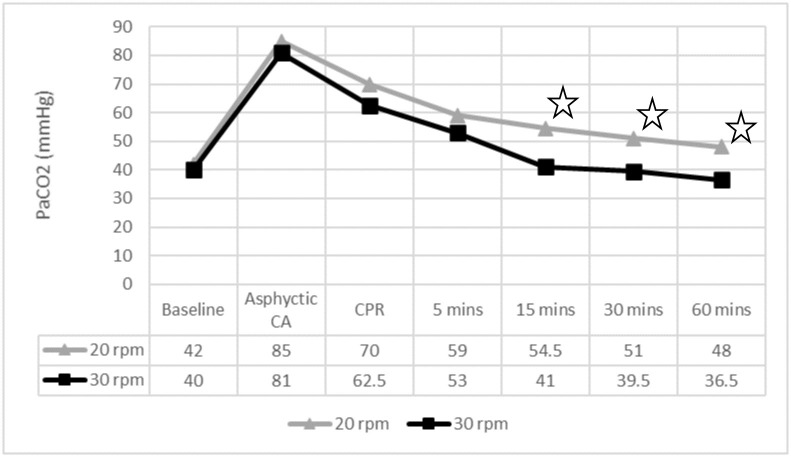
PaCO2 levels throughout the experiment. ✩ p < 0.05. PaCO2: CO2 arterial pressure; CA: cardiac arrest; CPR: cardiopulmonary resuscitation; mins: minutes. 5 mins to 60 mins: time after ROSC.

**Fig 3:**
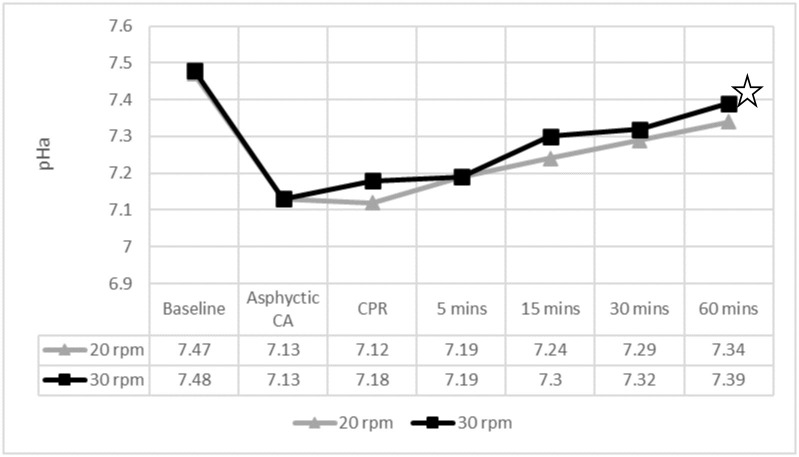
Arterial pH levels throughout the experiment. ✩ p < 0.05. pHa: arterial pH; CA: cardiac arrest; CPR: cardiopulmonary resuscitation; mins: minutes. 5 mins to 60 mins: time after ROSC.

Regarding ventilation, the 30 rpm group showed statistically lower PaCO2 levels than the group receiving 20 rpm after 15, 30 and 60 minutes of ROSC. Differences in arterial pH were only observed after 60 minutes of ROSC (Figs 2 and 3). The percentage of normoventilated piglets was significantly higher in the 30 rpm group during the entire study period (Table 4). Hyperventilation in the 30 rpm group was only observed in one piglet, 60 minutes after ROSC.

**Table 4:** Normoventilated piglets (PaCO2 30–50 mmHg) after ROSC.

Time post-ROSC	Normoventilated N/total (%)		p-value
	20 rpm	30 bpm	
5 minutes	2/25 (8%)	12/25 (48%)	**0.02**
15 minutes	5/24 (20.8%)	19/21 (90.5%)	**< 0.01**
30 minutes	12/25 (48%)	22/24 (91.7%)	**< 0.01**
60 minutes	16/25 (64%)	21/23 (91.3%)	**0.02**

Data are expressed as medians (IQR). Statistically significant values (p <0.05) appear in bold type.

ROSC: return of spontaneous circulation.

**Fig 4:**
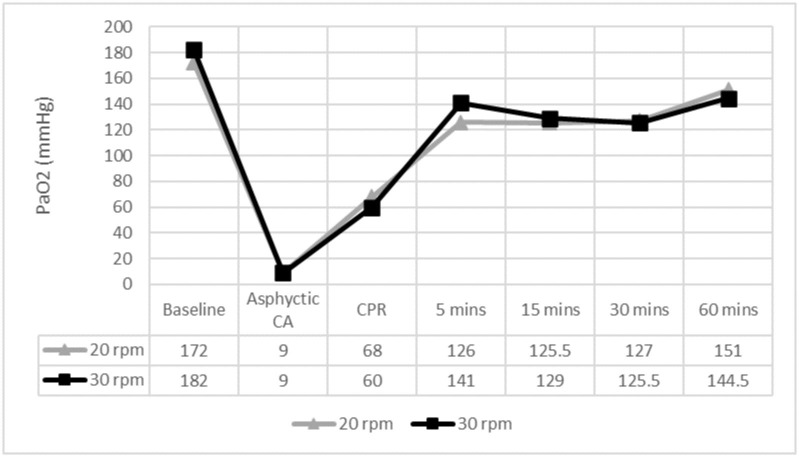
PaO2 levels throughout the experiment. ✩ p < 0.05. PaO2: O2 arterial pressure; CA: cardiac arrest; CPR: cardiopulmonary resuscitation; mins: minutes. 5 mins to 60 mins: time after ROSC.

**Fig 5:**
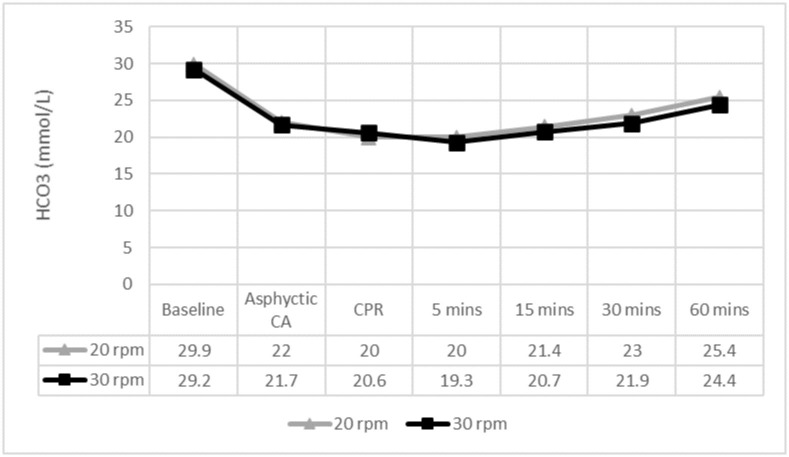
HCO3 levels throughout the experiment. ✩ p < 0.05. HCO3: bicarbonate; CA: cardiac arrest; CPR: cardiopulmonary resuscitation; mins: minutes. 5 mins to 60 mins: time after ROSC.

No differences were observed in PaO2 or HCO3 between groups (Figs 4 and 5).

## Discussion

This is the first prospective study to analyze the differences in hemodynamic, perfusion and ventilation parameters between two different ventilation strategies during the first hour after ROSC in a pediatric animal model of CA. This animal model could serve as a basis for future experimental studies on the effect of ventilation and oxygenation in the immediate period after ROSC.

Delivering 30 rpm improved ventilation within the first 60 minutes after ROSC, without a negative impact on hemodynamics or cerebral perfusion. In the light of these results, perhaps children would also benefit from higher respiratory rates during the first hour after ROSC, until normoventilation is achieved.

Hyperventilation after ROSC has been associated with poor outcomes. Nevertheless, the deleterious effect of hyperventilation in these studies refers to that occurring after 24–48 hours of ROSC. In these studies, the earliest registered parameters were taken, at least, 60 minutes after ROSC. This is the first study to assess the effects of ventilation in the immediate period (within the first 60 minutes) after ROSC [4,15].

Ventilation within these ranges of RR seems to be safe, as no differences were found in hemodynamic parameters between groups. These results are broadly consistent with those reported by other studies [12,17].

Lactate levels were elevated at ROSC and gradually decreased within the following 60 minutes in both groups. Lactate clearance was slightly higher in the 20 rpm group (3.8 mmol/L vs 3.3 mmol/L). This difference, was not clinically relevant as the remaining perfusion parameters were normal in the 30 rpm group.

Lactate is a good marker of tissue hypoxia and is widely used to assess tissue perfusion in critically ill children after CA [12,18,19]. A rapid decrease in lactate levels is an indicator of good prognosis [18]. However, lactate levels can remain elevated after CA despite an adequate tissue perfusion due to the administration of epinephrine or the presence of hyperglycemia [1,4].

PaCO2 is a determining factor in cerebral perfusion, as it regulates cerebral blood flow [20]. Hypercapnia increases and hypocapnia reduces cerebral blood flow [21]. A reduced cerebral blood flow may worsen cerebral ischemia [2,14,22–24]. Despite the differences in PaCO2, no significant differences were found in cerebral oxygenation or carotid blood flow, except in cerebral rSO2 at 5 minutes post-ROSC.

PaCO2 < 30 mmHg and > 50 mmHg at 4 hours and 24 hours post-ROSC seem to be indicators of mortality [1,4]. This is why normoventilation was defined as PaCO2 between 30–50 mmHg in our study.

The 30 rpm group had a significantly lower PaCO2 and reached normoventilation faster than the 20 rpm group, which remained hypoventilated until the end. Only one piglet in the 30 rpm group was hyperventilated at the end of the observation period.

Arterial pH was higher in the 30 rpm group, but significant differences were only found at 60 minutes after ROSC. No differences were observed in terms of oxygenation, as reported in other studies [7].

Our study has several limitations. In the first place, these results must be taken with caution due to the experimental nature of the study. In the second place, the study period was limited to a very short period after ROSC. What happens in these first 60 minutes after ROSC may be crucial, but not the only factor determining patient´s outcome. Future research should consider the potential effects of ventilation during the first 60 minutes after ROSC, and combine them with what happens in the following 24 to 48 hours, in order to provide an integrated perspective of different ventilation strategies and their impact on outcome.

## Conclusions

A respiratory rate of 30 rpm during the first hour after ROSC is more effective to achieve normoventilation than 20 rpm in a pediatric animal model of asphyxial CA. This ventilation strategy seems to be safe, as it does not cause hyperventilation and doesn’t affect hemodynamics or cerebral tissue perfusion.

## Supporting information

S1 Data (SAV)
